# Molecular mechanisms of action of trypanocidal and leishmanicidal drugs with focus on novel chemotherapeutic strategies: creation of a Brazilian multicentre working group

**DOI:** 10.1590/0074-02760220002

**Published:** 2022-05-06

**Authors:** Rubem Figueiredo Sadok Menna-Barreto, André Luis Souza dos Santos

**Affiliations:** 1 Fundação Oswaldo Cruz, Rio de Janeiro, Brasil; Universidade Federal do Rio de Janeiro, Rio de Janeiro, Brasil

Brazilian scientists are pioneers and leaders in diverse fields involving studies on *Trypanosoma cruzi* and *Leishmania* spp., which are flagellated trypanosomatids etiological agents of well-recognised neglected tropical diseases called Chagas disease (CD) (American trypanosomiasis) and leishmaniases (cutaneous, mucocutaneous and visceral forms), respectively, that afflict many countries in the world, particularly Brazil, with impressive impact in human health. Nowadays, in the 21st century, it seems somewhat unbelievable that these neglected tropical diseases continue to present unacceptable and frightening morbimortality rates. Actually, these diseases do not receive the veritable attention that should be given by the public and private health authorities, resulting in deaths that could be avoided.

According to the World Health Organization (WHO), CD is found mainly in endemic areas of 21 continental Latin American countries, with an estimated 6 to 7 million people worldwide infected with *T. cruzi*. Regarding *Leishmania*, 92 and 83 countries/territories are considered endemic for cutaneous and visceral leishmaniasis, respectively, with an estimated 30,000 new cases of visceral leishmaniasis and more than 1,000,000 new cases of cutaneous leishmaniasis per year. To worsen the current situation, estimates reveal that more than 1 billion people live in areas endemic for leishmaniasis and are at risk of infection.

Leishmaniasis and CD share a common problem: few drugs are clinically available. The current treatments for both diseases have doubtful efficacy and require continuous monitoring. Moreover, the drug arsenal against CD (benznidazole and nifurtimox) and leishmaniasis (pentavalent antimonial agents, amphotericin B, pentamidine and miltefosine) presents several problems regarding their administration (e.g., need for hospitalisation), long-time usage, the appearance of several and severe side effects and the emergence of parasite resistance. Therefore, the innovations in therapeutic strategies and novel or “rediscovered” promising candidates to efficacious combat leishmaniasis and CD are urgently required.

In this scenario, the workshop “Molecular mechanisms of action of trypanocidal and leishmanicidal drugs” was organised in September 2021, and it was transmitted by Oswaldo Cruz Institute channel in the YouTube platform (https://www.youtube.com/watch?v=4_2_wclBpJQ). The event consisted of 14 lectures of researchers from different Brazilian Institutions (Instituto Oswaldo Cruz, Universidade Federal do Rio de Janeiro, Instituto Adolfo Lutz, Centro de Pesquisas René Rachou, Universidade Federal de Minas Gerais, Universidade de São Paulo, Universidade Estadual de Maringá) with proven expertise in the field, focused in discussing the limitations of the current treatment as well as new targets and/or therapeutic strategies that should be further addressed. Visit the website to see the video presentations. The online event audience included students from different academic levels, researchers, teachers, technicians, reaching almost 2,500 views. In two days, many relevant aspects were raised, among which a proposal of interactive networks emerged as an urgent necessity.

Based on this premise, after the end of the Meeting, the researchers were invited by the hosts to stress and to discuss in more detail the ideas as well as the needs that arose during the event in order to evolve the pertinent agenda on new strategies against CD and leishmaniasis. In the assembly, the International Consortium of Trypanocidal and Leishmanicidal Drugs (ICTLD) was consolidated and some priority activities were proposed by the group. Among the first activities, the creation of pipelines for the screening of active drugs for CD, leishmaniasis and immunomodulators (for both diseases) was a main concern. In parallel, a pipeline of targets and hits identification (including *in silico* steps) will be also prioritised. With the complete chart organised, mapping the biological, chemical and human resources available (with a creation of a bank of bioactive molecules), the goals of the ICTLD will be determined based on One Health approach. Relevantly, the ICTLD aims to promote educational and training workshops as well as scientific seminars and meetings in order to disseminate the knowledge in the field. Also, ICTLD will lobby various agencies to increase funding for translational researchers and treatments of these Brazilian endemic parasitic infections.

To celebrate the event and the creation of the ICTLD, 13 perspective articles were written by different Brazilian specialists, discussing promising alternative therapeutic approaches/strategies to combat CD and leishmaniasis. In this context, the perspective written by Soeiro strengthened the necessity of the development of novel therapeutic options for CD based on the limitations of the current clinical compounds: particularly the low efficacy in the chronic stage, important side effects and variable efficacy against some parasite strains. She also briefly revised the data on phosphodiesterase inhibitors as new compounds against *T. cruzi*. Sangenito and colleagues stressed the drug repurposing as a promising strategy against CD, describing the potent effects of aspartyl peptidase inhibitors used in the anti-HIV therapy against the clinically relevant forms of *T. cruzi*. Ennes-Vidal and colleagues also proposed peptidases from these parasites as interesting and viable targets; however, the authors focused on calpains, which are calcium-dependent cysteine peptidases highly expanded in the genome of the trypanosomatids with participation in several key biological processes. Andrade-Neto and colleagues pointed to the uptake of host cholesterol by *Leishmania* species as a possible drug strategy, suggesting that cholesterol plays an essential role in the biological activity of sterol biosynthesis inhibitors. Monte-Neto and colleagues reviewed the recent advances on the antileishmanial metallodrugs as well as the elucidation of drug targets based on post-translational modifications.

Energetic and oxidative metabolisms stand out among the most recurrent drug targets in trypanosomatids. Pedra-Rezende and colleagues highlighted the mitochondrion of the parasites, which is a vital and unique organelle, as an excellent target to the action of novel synthetic compounds. Santi and Murta further discussed the antioxidant machinery of trypanosomatids as a potential target for chemotherapy, focusing on iron superoxide dismutase A, tryparedoxin peroxidase and ascorbate peroxidase as well as their involvement with drug resistance mechanisms in these parasites. Lazarin-Bidóia and colleagues described several Brazilian plant extracts as well as isolated compounds from natural sources as potent drugs able to interfere with the physiology of the unique mitochondrion of these parasites.

Dantas and colleagues revisited high-throughput campaigns for the screening of new active compounds against trypanosomatids, discussing perspectives of phenotypic approaches with emphasis on the role of image-based, high-content, methods, proposing an ideal cascade of assays for the identification of new drug candidates for clinical development. In this same way, Santos and Ferreira compiled the most recent data about the computational approaches for the design of potent and specific inhibitors against cruzain, the main multifunctional cysteine peptidase of *T. cruzi*.

In relation to immunomodulatory therapies, Waghabi and colleagues highlighted the possible use of TGF-β as an effective strategy and a therapeutic target to improve cardiac function with potential heart regeneration. Lannes-Vieira reinforced, based on preclinical trials using hypothesis-based tools, the opened paths for multi-therapeutic approaches targeting parasite and inflammation-related alterations in chronic chagasic cardiomyopathy. Finally, Araújo-Jorge and Ferreira discussed the translational research approaches for the development of alternatives for neglected diseases including CD, based on the experience in selenium trials. Among the perspectives of our future Consortium is a complementary clinical trial for the opened questions for these diseases.

The editors of this special issue of *Memórias do Instituto Oswaldo Cruz* believe that the proposed theme will be of great interest to the scientific community, which is a dynamic, effervescent, contemporary and extremely relevant discussion on this field. In this context, the editors would like to take this opportunity to thank all the researchers who contributed with excellent and up to date perspectives on the search for novel chemotherapeutic strategies against leishmaniasis and CD. Last but not least, the editors would like to deeply express their gratitude to all the researchers who helped in the meticulous process of review of the articles comprising this special volume, particularly to Dr Marta Helena Branquinha (Universidade Federal do Rio de Janeiro - Instituto de Microbiologia Paulo de Góes) and Dr Solange de Castro (Fundação Oswaldo Cruz - Instituto Oswaldo Cruz), as well as the members forming the editorial office of *Memórias do Instituto Oswaldo Cruz*, who are extremely compromised, efficient and competent staff.

The editors believe that operating tools and strategies to stimulate the search for new antimicrobial drugs are imperative tasks and should continue to be constantly applied and advanced, since these initiatives will not happen spontaneously. Finally, the editors really hope that the reading of each review and, of course, the special issue as a whole, will arouse enthusiasm and scientific curiosity in young students and researchers around the world.

